# Impairment and Disability Identity and Perceptions of Trust, Respect, and Fairness

**DOI:** 10.1001/jamahealthforum.2023.3180

**Published:** 2023-09-22

**Authors:** Maggie R. Salinger, Brian Feltz, Stephanie H. Chan, Anna Gosline, Carine Davila, Suzanne Mitchell, Lisa I. Iezzoni

**Affiliations:** 1 Division of General Internal Medicine, Massachusetts General Hospital, Boston, Massachusetts; 2 3D Research Partners LLC, Harvard, Massachusetts; 3 Flowetik, Boston, Massachusetts; 4 Massachusetts Coalition for Serious Illness Care, Boston, Massachusetts; 5 Blue Cross Blue Shield of Massachusetts, Boston, Massachusetts; 6 Division of Palliative Care and Geriatric Medicine, Massachusetts General Hospital, Boston, Massachusetts; 7 University of Massachusetts Medical School, Worcester, Massachusetts; 8 Health Policy Research Center, Massachusetts General Hospital, Boston, Massachusetts; 9 Harvard Medical School, Boston, Massachusetts

## Abstract

**Importance:**

Most studies use impaired functioning alone to specify populations with disabilities. However, some people with functional impairments do not identify as disabled. With functional status-based definitions, studies have shown disparate care quality for people with disabilities.

**Objective:**

To examine whether impairment and disability identity have different associations with perceived health care experiences and explore factors associated with disability identification.

**Design, Setting, and Participants:**

This cross-sectional study used a nationally representative survey of US adults conducted from April 20 through May 31, 2021, and analyzed between June 1 and August 31, 2022. Survey participants were 1822 English- or Spanish-speaking adults responding either online or via telephone.

**Exposures:**

Using 8 survey questions, participants were grouped according to presence of impairment and disability identity.

**Main Outcomes and Measures:**

Likert scale measures of trust, respect, and fairness (henceforth, procedural justice measures) were dichotomized. Sociodemographic characteristics and rates of procedural justice responses were compared across groups. Multivariable logistic regressions adjusting for baseline characteristics were performed to (1) estimate associations of impairment and disability identity with perceptions of procedural justice and (2) explore factors associated with disability identification. Analyses applied survey weights.

**Results:**

Of 6126 individuals invited to participate, 1854 (30.3%) completed the survey. Thirty-two were excluded due to unreportable gender, for a final analytic sample of 1822 participants. Participants with impairments (n = 816; mean [SD] age, 48.1 [17.0] years; 51.2% women, 48.8% men) had worse perceptions on 7 of 10 procedural justice measures (crude) compared with those without impairments (n = 1006; mean [SD] age, 49.6 [18.1] years, 55.1% female, 44.9% male). Among respondents with impairments, those who did (n = 340) vs did not (n = 476) identify as disabled gave better ratings for clinician communication efforts (a lot of effort, 38.8% vs 31.0%) and having health goals understood (understood very or fairly well, 77.2% vs 70.1%) but gave worse ratings for respect (almost never felt inferior or talked down to, 66.1% vs 59.1%). Disability identification was associated with more reports of unfair treatment (31.0% vs 22.4%; adjusted odds ratio, 1.65; 95% CI, 1.12-2.42) and of being unafraid to ask questions or disagree (50.5% vs 40.1%; adjusted odds ratio, 1.51; 95% CI, 1.04-20.19). Income and employment were associated with disability identification.

**Conclusions and Relevance:**

In this cross-sectional survey study of US adults, health care perceptions differed between groups defined by impairment status and disability identity. These findings suggest that, alongside functional measures, health systems should capture disability identity to better address disparities for people with impairments.

## Key Points

**Question:**

Are impairment and disability identity associated with patient perceptions of procedural justice (ie, trust, communication, respect, and fairness) in health care encounters?

**Findings:**

In this cross-sectional analysis of 1822 survey participants, participants with impairments had worse perceptions of procedural justice in health care visits than those without impairments. Participants with functional impairments who identified as disabled rated clinicians’ effort and understanding of health goals more favorably but their fairness and respectful communication worse than participants without disability identity.

**Meaning:**

These findings suggest that alongside functional measures, health systems should capture disability identity to better address disparities for patients with disabilities.

## Teaser

This cross-sectional study examines the association of impairment and disability identity with perceptions of procedural justice among adults with disabilities living in the US.

## Introduction

More than 1 in 4 community-dwelling US adults have a disability based on measures of impaired function.^1^ A substantial and growing body of literature documents health care inequities that affect people with disabilities.^2,3,4,5,6,7,8,9^ Compared with adults without disabilities, those with disabilities are almost twice as likely to delay seeking care due to cost, to be unemployed or in the lowest income brackets, and to face transportation barriers.^2,4,10^ This population also has higher rates of smoking, obesity, and incident diabetes along with lower rates of up-to-date mammograms and Papanicolaou tests.^2,5,6,7,8^ Research has further shown that people with disabilities are less likely to receive definitive treatment for breast cancer and have higher rates for all-cause and disease-specific mortality.^9^

Most medical schools provide little training about disability.^11,12,13,14,15,16,17^ The resultant gap in awareness and skills is compounded by bias (implicit or explicit) and by misconceptions about quality of life of people with disabilities.^18^ In a nationwide survey of US physicians, 35.8% knew little or nothing about their legal obligations under the Americans With Disabilities Act, and only 40.7% felt very confident that they could provide the same quality of care to patients with disabilities as they do for patients without disabilities.^14,15^ In this same survey, 82.4% of physicians conflated disability with a lower quality of life.^15^

The growing attention to disability disparities has coincided with an increasing recognition of a need to capture high-quality data.^3,19,20,21,22^ Section 4302 of the Affordable Care Act calls for a minimum standardized measure of disability in all federally funded surveys.^21,22^ The chosen method was a series of 6 yes-no questions from the American Community Survey (ACS-6) that ask about serious difficulty with the following basic functions: seeing; hearing; remembering, making decisions, or concentrating; ambulating; caring for self; and/or doing errands. At least 1 “yes” answer indicates disability.^22,23^

Despite the value of standardizing disability questions, the chosen approach has several limitations, including low sensitivity and inconsistent results.^3,20,24,25^ The chosen standards also situate disability within the individual through their exclusive focus on impairments in basic function, even though disability is mediated by environmental factors and can manifest as limitations in other unmeasured forms of societal participation, such as work, hobbies, or social activities.^26^

In addition, the standard disability questions do not ask about identity. The degree of overlap between individuals who are considered to have a disability by their ACS-6 responses and those who self-identify as disabled is unknown and may vary by other personal or cultural attributes.^27,28,29,30^ Disability identity is a key dimension of diversity that is distinct from one’s impairment status and that “shapes a person’s ways of seeing themselves, their bodies, and their way of interacting with the world.”^28^^(p198)^ Furthermore, some factors that shape identity also are associated with health injustices and disparities, including stigma, bias, politics, and systems of power or oppression.^31^

This study analyzes findings from a nationally representative survey to explore which sociodemographic factors could be associated with the adoption of a disability identity and to examine whether impairment status and disability identity are associated with patients’ perceptions of trust, communication, respect, and fairness in clinical encounters. Henceforth, these concepts are considered components of procedural justice, a type of justice that is concerned with perceptions about processes that correlate with views of both situational outcomes and institutional legitimacy.^32,33,34^ We hypothesized that disability identity is independently associated with procedural justice measures, even when controlling for other sociodemographic traits. Recognizing different preferences, we use both person-first and identity-first disability language throughout.

## Methods

This cross-sectional study is part of a larger study by the Massachusetts Coalition for Serious Illness Care (MCSIC), which commissioned a survey conducted between April 20 and May 31, 2021, to learn about patients’ perceptions of health care. The NORC (National Opinion Research Center) institutional review board oversaw survey recruitment, administration, and informed consent. The Harvard Longwood Campus institutional review board approved the survey instrument and analysis. We followed the American Association for Public Opinion Research (AAPOR) reporting guidelines.

### Study Sample and Survey Instrument

NORC at the University of Chicago recruited a nationally representative sample of 1854 adults (aged ≥18 years) via AmeriSpeak, its probability-based household panel with 97% coverage of the residential US.^35^ Enrollment targets were set for vulnerable groups (determined by self-selected race, self-selected ethnicity, age, impairments, and income) (eMethods and eFigure 1 in Supplement 1). NORC staff collected data online or by telephone, in English or Spanish.

**Figure 1.**
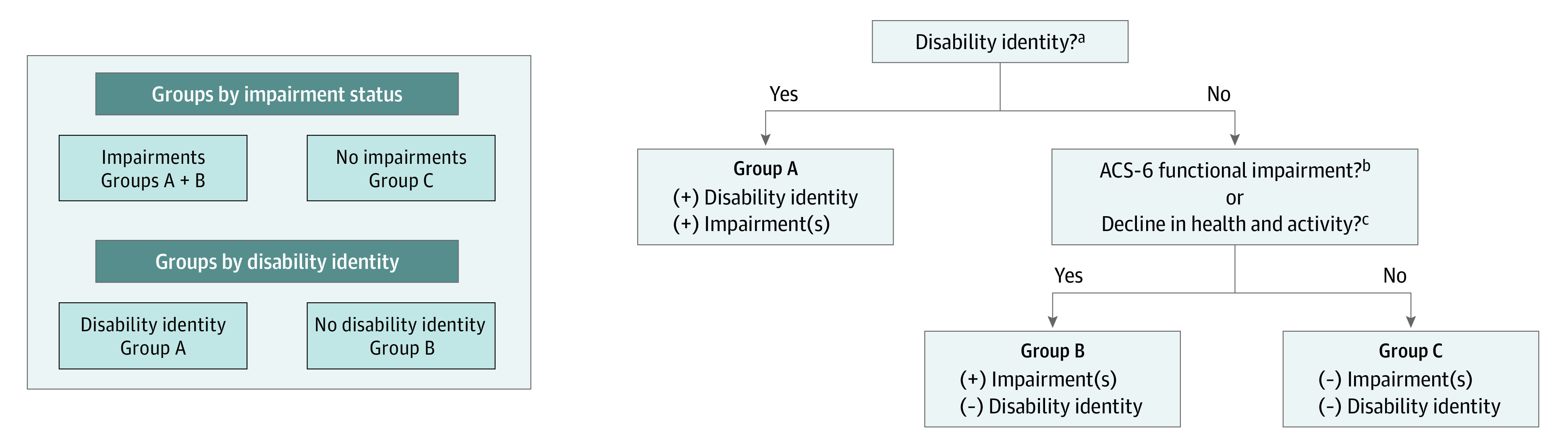
Methods for Grouping Survey Respondents by Impairment Status and Disability Identity ^a^Disability identity was determined by the question, “Do you have a disability?” ^b^Functional impairment status refers to responses for 6 questions that were derived from the American Community Survey (ACS-6): (1) “Are you deaf, or do you have serious difficulty hearing?” (2) “Are you blind, or do you have serious difficulty seeing, even when wearing glasses?” (3) “Because of a physical, mental, or emotional condition, do you have serious difficulty concentrating, remembering, or making decisions?” (4) “Do you have serious difficulty walking or climbing stairs?” (5) “Do you have difficulty dressing or bathing?” (6) “Because of a physical, mental, or emotional condition, do you have difficulty doing errands alone, such as visiting a doctor’s office or shopping?” ^c^Decline in health and activity was assessed via the question, “Over the past 12 months, would you say that you have been feeling sicker and that it has been getting harder to do your normal levels of work and activity?”

The survey asked 7 impairment-focused questions (ACS-6 plus a reported decline in health and activity) and 1 question that approximated disability identity by asking, “Do you have a disability?” (for a total of 8 yes-no questions). We grouped participants using these 8 questions (Figure 1) and categorized those who responded no to all 8 as having no impairment and the rest as having an impairment. Among respondents with impairments, we further divided them according to those who indicated having a disability (Yes-DAI group) and those who said no to having a disability but yes to 1 or more of the 7 impairment-focused questions (No-DAI group). Next, we dichotomized Likert scale measures of procedural justice according to the distribution of responses and meaning of the answers (eTable in Supplement 1).

### Statistical Analysis

We conducted analyses from June 1 to August 31, 2022. Demographic categories were self-reported in the survey. Due to small size, several genders were unreportable: transgender male (n = 5), transgender female (n = 10), nonbinary (n = 7), and other or skipped the question (n = 10). To avoid confounding, we excluded these 32 respondents rather than forming a heterogeneous group. Asian (n = 49), multiracial (n = 68), and other (n = 34) racial categories also had a small cell size across groups but were collapsed since they are not known confounders. Using χ^2^ for categorical variables and *t* test and ANOVA for continuous variables, we compared sociodemographic indicators and rates of dichotomized procedural justice responses across groups. We performed multivariable logistic regressions adjusting for baseline characteristics to estimate associations of impairment status and disability identity on procedural justice perceptions. We also examined factors associated with disability identification among participants with impairments using nested multivariable logistic regression models.

Covariates included baseline sociodemographic traits that were unevenly distributed. We excluded insurance status because of its collinearity with the exposure variable (group) and other sociodemographic indicators (eg, age and income).

In analyses among participants with impairments, we further adjusted models for type and number of impairments (maximum, 7: ACS-6 plus decline in health and activity, dichotomized to ≤3 vs ≥4 due to cell size). We did not control for hearing or visual impairments, since these were evenly distributed.

Due to the exploratory nature of our study, we maintained a significance threshold of *P* < .05 (2-sided). All analyses applied survey weights to reflect the US census population and were performed using SPSS, version 29 statistical software (IBM Corporation).

## Results

Of the 6126 AmeriSpeak panelists invited to participate, 1854 (30.3%) completed the MCSIC survey. Excluding the 32 individuals with unreportable gender, the final analytic sample included 1822 individuals. The margin of error was 3.1 percentage points, adjusted for design effect.^1,36^

### Impairments vs No Impairments

#### Baseline Characteristics

As shown in Table 1, compared with participants without impairments (n = 1006; mean [SD] age, 49.6 [18.1] years, 55.1% women, 44.9% men, 14.4% Black, 15.8% Hispanic, 69.4% White, 16.2% other race), those with impairments (n = 816; mean [SD] age, 48.1 [17.0] years; 51.2% female, 48.8% male, 11.8% Black, 17.1% Hispanic, 72.7% White, 15.5% other race) were less likely to be working (49.3% vs 67.0%) or married or partnered (58.2% vs 65.5%) and more likely to have the lowest income (<$30 000 per year, 24.4% vs 12.5%) or educational attainment (high school or less, 41.4% vs 33.8%). Additionally, the impairment group was more likely to have Medicare (24.1% vs 16.1%) or Medicaid (16.0% vs 5.1%).

**Table 1.** Baseline Characteristics of Survey Respondents, Categorized by Presence of Impairment and Disability Identity

	Total	No. (%)^a^					
		Impairments?^b^			Disability identity?^b^		
		Yes	No	*P* value^c^	Yes	No	*P* value^c^
No. of respondents	1822	816	1006		340	476	
Age, mean (SD), y	48.7 (17.5)	49.6 (18.1)	48.1 (17.0)	<.001	53.7 (16.9)	47.3 (18.3)	.06
Age category, y							
18-34	466 (28.7)	191 (28.5)	275 (28.8)	.08	53 (17.5)	138 (34.7)	<.001
35-54	538 (30.3)	230 (28.7)	308 (31.4)		99 (31.0)	131 (27.4)	
55-74	675 (34.2)	317 (34.3)	358 (34.1)		157 (42.3)	160 (29.7)	
≥75	143 (6.9)	78 (8.6)	65 (5.7)		31 (9.2)	47 (8.3)	
Gender							
Men	867 (47.1)	372 (44.9)	495 (48.8)	.10	155 (42.2)	217 (46.5)	.25
Women	955 (52.9)	444 (55.1)	511 (51.2)		185 (57.8)	259 (53.5)	
Race							
Black	203 (12.9)	98 (14.4)	105 (11.8)	.20	52 (18.0)	46 (12.4)	.03
White	1360 (71.3)	593 (69.4)	767 (72.7)		235 (69.2)	358 (69.5)	
Other^d^	259 (15.8)	125 (16.2)	134 (15.5)		53 (12.9)	72 (18.1)	
Hispanic ethnicity	279 (1.6.6)	130 (15.8)	149 (17.1)	.49	50 (14.1)	80 (16.8)	.34
Marital status							
Married or living with partner	1078 (62.4)	459 (58.2)	619 (65.5)	.01	163 (48.8)	296 (63.9)	<.001
Divorced or separated	297 (13.6)	153 (15.2)	144 (12.4)		76 (20.0)	77 (12.4)	
Widowed	67 (3.1)	35 (3.8)	32 (2.5)		21 (5.5)	14 (2.9)	
Never married	380 (20.9)	169 (22.8)	211 (19.5)		80 (25.7)	89 (21.1)	
Annual household income							
<$30 000	424 (17.6)	250 (24.4)	174 (12.5)	<.001	139 (36.2)	111 (17.7)	<.001
$30 000 to <$60 000	519 (25.5)	250 (28.1)	269 (23.5)		111 (29.7)	139 (27.2)	
$60 000 to <$100 000	457 (30.4)	172 (27.9)	285 (32.2))		53 (22.1)	119 (31.1)	
≥$100 000	422 (26.6)	144 (19.6)	278 (31.7)		37 (12.0)	107 (24.0)	
Education level							
High school or less	366 (37.0)	198 (41.4)	168 (33.8)	<.001	95 (46.5)	103 (38.5)	.01
Some college	779 (27.7)	362 (29.2)	417 (26.6)		163 (31.4)	199 (27.9)	
Bachelor’s degree	381 (19.8)	134 (15.1)	247 (23.3)		40 (11.9)	94 (16.8)	
Graduate or professional degree	296 (15.4)	122 (14.4)	174 (16.2)		42 (10.4)	80 (16.7)	
Employment status							
Working (full or part time)	1059 (59.4)	376 (49.3)	683 (67.0)	<.001	96 (28.7)	280 (61.1)	<.001
Unemployed (laid off or seeking work)	98 (7.0)	52 (6.9)	46 (7.1)		19 (5.1)	33 (7.9)	
Not working (disabled or other)	246 (12.8)	179 (20.5)	67 (7.1)		134 (38.6)	45 (10.2)	
Retired	419 (20.7)	209 (23.3)	210 (18.8)		91 (27.6)	118 (20.8)	
Insurance status							
Not insured	182 (11.4)	85 (11.9)	97 (11.0)	<.001	23 (7.8)	62 (14.3)	<.001
Medicare	408 (19.5)	233 (24.1)	175 (16.1)		131 (36.0)	102 (17.3)	
Medicaid	179 (9.8)	121 (16.0)	58 (5.1)		62 (20.0)	59 (13.8)	
Private	931 (52.5)	315 (41.4)	616 (60.8)		92 (28.5)	223 (48.7)	
Other	122 (6.8)	62 (6.5)	60 (7.0)		32 (7.7)	30 (5.8)	
Geographic region							
Northeast	252 (17.4)	102 (15.2)	150 (19.1)	.18	42 (12.6)	60 (16.6)	.40
Midwest	486 (20.9)	218 (21.3)	268 (20.6)		90 (23.5)	128 (20.0)	
South	617 (37.8)	283 (39.4)	334 (36.6)		114 (40.5)	169 (38.8)	
West	467 (23.9)	213 (24.1)	254 (23.7)		94 (23.3)	119 (24.6)	
Caregiver status^e^	388 (20.4)	215 (27.1)	173 (15.3)	<.001	95 (27.6)	120 (26.9)	.81
Veteran or active military	155 (6.6)	85 (7.3)	70 (6.1)	.27	48 (10.4)	37 (5.6)	.02
Answered yes to >1 ACS-6 question	488 (23.6)	NA	NA	NA	209 (60.8)	239 (51.9)	.02
Impairment types							
Hearing	95 (4.3)	NA	NA	NA	33 (8.8)	62 (10.8)	.39
Vision	75 (5.0)	NA	NA	NA	41 (13.6)	34 (10.8)	.38
Cognitive or psychological	197 (10.5)	NA	NA	NA	101 (29.0)	96 (22.0)	.03
Mobility	187 (9.4)	NA	NA	NA	120 (35.6)	67 (14.3)	<.001
Dressing or bathing	43 (1.9)	NA	NA	NA	32 (8.1)	11 (2.5)	<.001
Errands	137 (7.0)	NA	NA	NA	89 (25.5)	48 (11.2)	<.001
Decline in health or activity	507 (26.9)	NA	NA	NA	178 (52.0)	329 (69.3)	<.001
Total impairments^f^							
≤3	756 (39.6)	NA	NA	NA	290 (85.7)	466 (97.4)	<.001
≥4	60 (2.9)	NA	NA	NA	50 (14.3)	10 (2.6)	
No. of impairments, mean (SD)^f^	0.65 (1.0)	NA	NA	NA	1.7 (1.3)	1.4 (0.8)	<.001

Abbreviations: ACS-6, standard set of 6 disability questions derived from the American Community Survey; NA, not applicable.

^a^ Baseline characteristics were self-reported in the survey. All analyses applied survey weights.

^b^ The impairments group was defined as respondents who identified as disabled, who reported a decline in health and activity, or who replied yes to any of the 6 functional limitation questions derived from the ACS-6. The no impairment group consisted of respondents who met none of the above criteria. Among respondents with impairments, subgroups of disability identity status were determined by yes vs no responses to the question, “Do you have a disability?”

^c^ The *P* values were derived from χ^2^ analyses for categorical variables and *t* test and analysis of variance for continuous variables.

^d^ The other racial category consists of respondents who selected Asian (n = 49), multiple race options (n = 68), or other (n = 34). These categories were collapsed due to small cell size across groups.

^e^ Caregivers were defined as those who indicated being very or fairly involved in someone else’s health care, such as through medical decision-making, care coordination, appointment-related transportation, provision of physical assistance, and billing and paperwork.

^f^ The maximum number of impairments is 7: 6 from the ACS-6 question set plus 1 question about decline in health and activity.

#### Procedural Justice Measures

**Figure 2.**
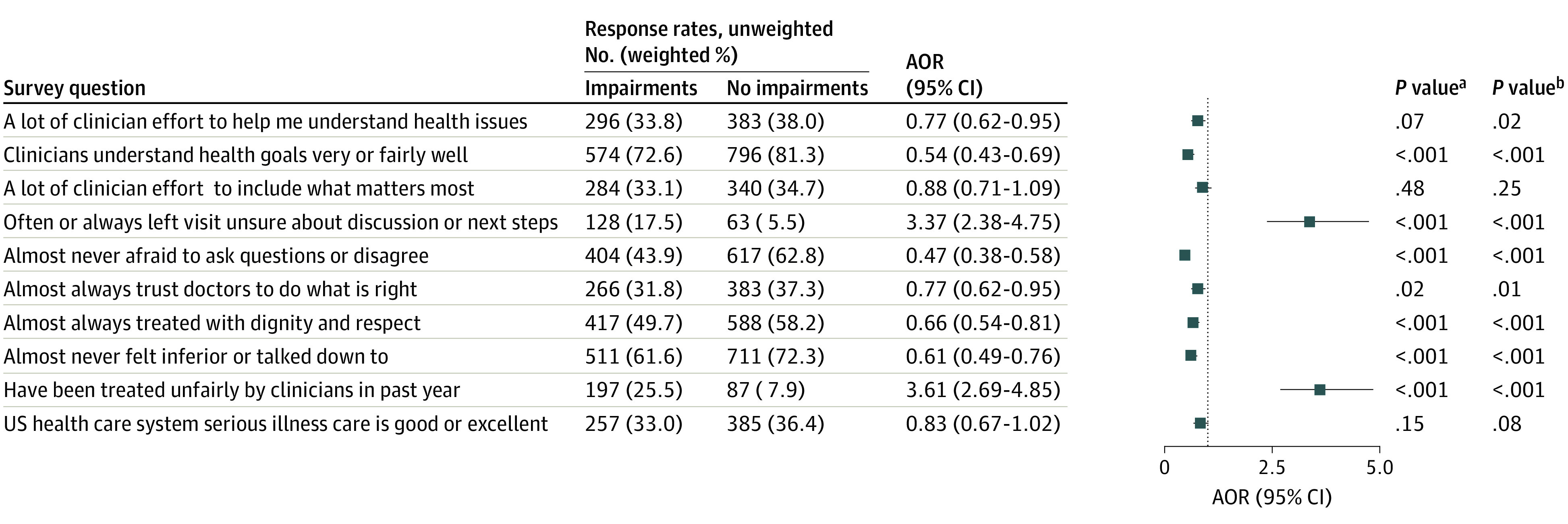
Dichotomized Procedural Justice Ratings Across All Respondents, Comparing Impairments vs No Impairments Responses to survey questions about procedural justice perceptions, ie, perceived trust, communication, respect, and fairness, were transformed from 5-point Likert scale measures to binary outcomes according to the distribution of responses and their valuative meaning. The impairments group was defined as respondents who identified as disabled, who reported a decline in health and activity, or who replied yes to any of the 6 functional limitation questions derived from the American Community Survey. The no impairment group consisted of respondents who met none of the above criteria. Logistic regression models were adjusted for age category, gender, race, ethnicity, education level, household income, employment status, marital status, caregiver status, and veteran or active military status. All covariates were based on survey self-report. All analyses applied survey weights. The number of survey respondents who skipped each question was 3, 5, 7, 2, 5, 2, 7, 11, 3, and 5 for rows 1 to 10, respectively. AOR indicates adjusted odds ratio. ^a^*P* value derived from χ^2^ analysis. ^b^*P* value derived from Wald test.

Figure 2 summarizes the proportions and the adjusted odds of dichotomized procedural justice ratings across all respondents and shows that compared with the no impairment group, the impairment group tended to have less favorable ratings of procedural justice in their health care experiences.

More than 1 in 6 respondents (17.5%) with impairments reported that they often or always felt unsure after their clinical encounters about the discussion or next steps compared with only 1 in 20 (5.5%) without impairments (adjusted odds ratio [AOR], 3.37; 95% CI, 2.38-4.75). Respondents with impairments also had significantly higher odds of perceiving unfair treatment by clinicians in the past year (AOR, 3.61; 95% CI, 2.69-4.85). Only 43.9% of the respondents with impairments said that they almost never felt afraid to ask questions or disagree with their provider compared with 62.8% without impairments (AOR, 0.47; 95% CI, 0.38-0.58).

### Disability Identity vs No Disability Identity

#### Baseline Characteristics

Table 1 shows that among respondents with impairments, 340 identified as having a disability (Yes-DAI group; mean [SD] age, 53.4 [16.9] years; 57.8% women and 42.2% men; 18.0% Black, 14.1% Hispanic, 69.2% White, and 12.9% other race), whereas 476 participants (mean [SD] age, 47.3 [18.3] years; 53.5% women and 46.5% men; 12.4% Black, 16.8% Hispanic, 69.5% White, and 18.1% other race) did not (No-DAI group). In the Yes-DAI group, 39.2% (n = 131) of participants indicated no impairment in response to the ACS-6 questions and, thus, would have been missed by this measure of function alone. In the No-DAI group, 51.9% (n = 239) were captured through the ACS-6 questions, with the remaining portion captured through the declining health and activity question. The Yes-DAI group had more total impairments (mean [SD], 1.7 [1.3] vs 1.4 [0.8]), whereas the No-DAI group had a higher prevalence of reported health decline (69.3% vs 52.0%). The Yes-DAI group had lower proportions of respondents who were married (48.8% vs 63.9%) or working (28.7% vs 61.1%) and higher proportions who were publicly insured (Medicare or Medicaid, 56.0% vs 31.1%), were in the lowest income bracket of<$30 000 per year (36.2% vs 17.7%), or had less than a college degree (77.9% vs 66.4%). The Yes-DAI group also had relatively more respondents who identified as Black (18.0% vs 12.4%) and who had a history of military service (10.4% vs 5.6%).

#### Procedural Justice Measures

**Figure 3.**
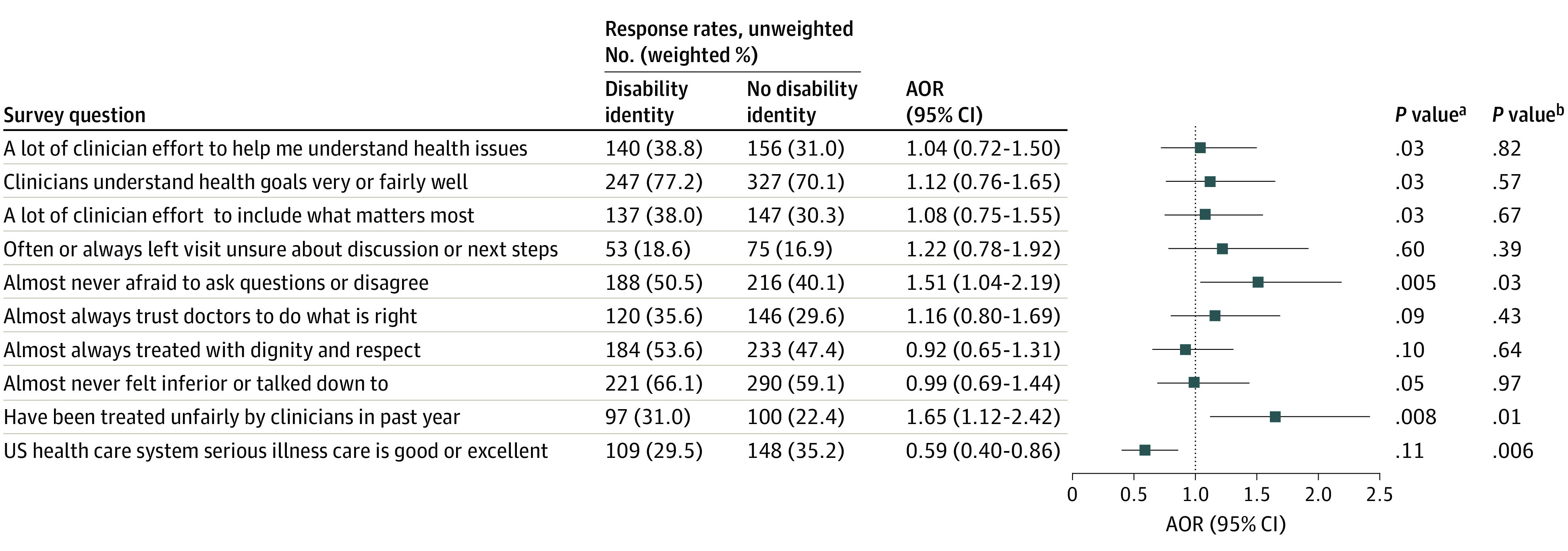
Dichotomized Procedural Justice Ratings Among Respondents With Impairments, Comparing Yes vs No Responses of Disability Identity Responses to survey questions about procedural justice perceptions, ie, perceived trust, communication, respect, and fairness, were transformed from 5-point Likert scale measures to binary outcomes according to the distribution of responses and their valuative meaning. The group with impairments was defined as respondents who identified as disabled, who reported a decline in health and activity, or who replied yes to any of the 6 functional limitation questions derived from the American Community Survey. Subgroups of disability identity status were determined by yes vs no responses to the question, “Do you have a disability?” Logistic regression models were adjusted for age category, gender, race, ethnicity, education level, household income, employment status, marital status, caregiver status, and veteran or active military status. All covariates were based on survey self-report. All analyses applied survey weights. Among respondents with impairments, the number who skipped each question was 1, 6, 1, 0, 1, 3, 2, 5, 0, and 0 for rows 1 to 10, respectively. AOR indicates adjusted odds ratio. ^a^*P* value derived from χ^2^ analysis. ^b^*P* value derived from Wald test.

Figure 3 lists the proportions and adjusted odds of dichotomized procedural justice ratings among respondents with impairments. Crude estimates show that many ratings were more favorable or similar for the Yes-DAI group compared with the No-DAI group. Respondents in the Yes-DAI group gave better ratings than those in the No-DAI group for clinician communication efforts (a lot of effort, 38.8% vs 31.0%) and having health goals understood (understood very or fairly well, 77.2% vs 70.1%), but gave worse ratings for respect (almost never felt inferior or talked down to, 66.1% vs 59.1%). Of respondents who identified as disabled, 50.5% reported that they were almost never afraid to ask questions or disagree with providers vs 40.1% of those with impairments who denied having a disability (AOR, 1.51; 95% CI, 1.04-2.19).

However, participants who identified as disabled had worse crude and adjusted fairness ratings. Among participants in the Yes-DAI group, 31.0% reported unfair treatment vs 22.4% of those in the No-DAI group (AOR, 1.65; 95% CI, 1.12-2.42). The Yes-DAI group also had a lower odds of rating serious illness care in the US as good or excellent (AOR, 0.59; 95% CI, 0.40-0.86). As shown in eFigure 2 in Supplement 1, the addition of covariates for number and type of impairments yielded similar AORs to those listed in Figure 3.

### Disability Identification

Table 2 summarizes the odds of adopting a disability identity among respondents with impairments, showing results from crude models as well as 2 adjusted models, including 1 that only controlled for sociodemographic traits and 1 that also adjusted for number and type of impairments. Gender, Hispanic ethnicity, and caregiver status were not associated with disability identity, while race and education were, though only in crude models. Wealthier respondents were less likely to identify as disabled in all models (highest [≥$100 000 per year] vs lowest<$30 000 per year] income: crude OR, 0.24 [95% CI, 0.15-0.39]; AOR model 1, 0.36 [95% CI, 0.19-0.68]; AOR model 2, 0.34 [95% CI, 0.18, 0.66]). Respondents who were unemployed for various reasons were more likely to identify as disabled than employed respondents across models (retired vs employed: crude OR, 2.82 [95% CI, 1.93-4.14]; AOR model 1, 6.33 [95% CI, 3.99-10.04]; AOR model 2, 4.94 [95% CI, 3.04-8.02]).

**Table 2.** Odds of Disability Identification Among Respondents With Impairments^a^

	Crude		Adjusted model 1^b^		Adjusted model 2^c^	
	OR (95% CI)	*P* value	AOR (95% CI)	*P* value	AOR (95% CI)	*P* value
Age category, y	NA	<.001	NA	<.001	NA	<.001
18-34	1 [Reference]	1 [Reference]	NA	1 [Reference]	1 [Reference]	NA
35-54	2.24 (1.48-3.39)	<.001	2.94 (1.77-4.87)	<.001	2.99 (1.75-5.11)	<.001
55-74	2.81 (1.89-4.19)	<.001	3.29 (1.87-5.79)	<.001	3.59 (1.93-6.65)	<.001
≥75	2.20 (1.22-3.95)	008	2.39 (1.03-5.55)	.04	2.41 (0.90-5.56)	.08
Female gender (reference male)	1.19 (0.89-1.60)	.25	1.12 (0.78-1.61)	.54	1.19 (0.81-1.73)	.38
Race	NA	.03	NA	.85	NA	.65
Black	1.45 (0.96-2.19)	.08	0.98 (0.60-1.62)	.95	0.80 (0.47-1.37)	.43
White	1 [Reference]	1 [Reference]	NA	1 [Reference]	1 [Reference]	NA
Other^d^	0.73 (0.47-1.09)	.12	0.87 (0.53-1.43)	.57	0.86 (0.52-1.43)	.56
Hispanic ethnicity	0.82 (0.54-1.23)	.33	1.13 (0.69-1.84)	.62	1.27 (0.76-2.10)	.36
Marital status	NA	<.001	NA	.01	NA	.02
Married or living with partner	1 [Reference]	1 [Reference]	NA	1 [Reference]	1 [Reference]	NA
Divorced or separated	2.11 (1.39-3.18)	<.001	1.21 (0.74-1.96)	.45	1.02 (0.61-1.71)	.93
Widowed	2.49 (1.18-5.26)	.02	1.32 (0.55-3.18)	.54	1.48 (0.60-3.66)	.40
Never married	1.58 (1.1-2.27)	.01	2.24 (1.39-3.61)	.001	2.21 (1.34-3.65)	.002
Annual household income	NA	<.001	NA	.005	NA	.007
<$30 000	1 [Reference]	1 [Reference]	NA	1 [Reference]	1 [Reference]	NA
$30 000 to <$60 000	0.53 (0.36-0.79)	.002	0.71 (0.45-1.14)	.16	0.74 (0.45-1.21)	.23
$60 000 to <$100 000	0.35 (0.23-0.52)	<.001	0.47 (0.28-0.78)	.003	0.49 (0.29-0.84)	.009
≥$100 000	0.24 (0.15-0.39)	<.001	0.36 (0.19-0.68)	.001	0.34 (0.18-0.66)	.001
Education level	NA	.01	NA	.50	NA	.42
High school or less	1 [Reference]	1 [Reference]	NA	1 [Reference]	1 [Reference]	NA
Some college	0.93 (0.66-1.3)	.69	1.36 (0.90-2.06)	.15	1.34 (0.87-2.06)	.18
Bachelor’s degree	0.59 (0.37-0.93)	.02	1.31 (0.75-2.29)	.35	1.54 (0.86-2.77)	.15
Graduate or professional degree	0.52 (0.32-0.83)	.006	1.11 (0.62-1.99)	.72	1.28 (0.7-2.33)	.43
Employment status	NA	<.001	NA	<.001	NA	<.001
Employed (full or part time)	1 [Reference]	1 [Reference]	NA	1 [Reference]	1 [Reference]	NA
Unemployed (laid off or seeking work)	1.36 (0.71-2.62)	.35	1.02 (0.50-2.07)	.97	1.08 (0.51-2.30)	.84
Not working (disabled or other)	8.01 (5.30-12.12)	<.001	2.06 (1.24-3.43)	.005	1.57 (0.92-2.68)	.10
Retired	2.82 (1.93-4.14)	<.001	6.33 (3.99-10.04)	<.001	4.94 (3.04-8.02)	<.001
Veteran or active military	0.51 (0.30-0.88)	.02	0.53 (0.28-1.00)	.05	0.50 (0.26-0.98)	.04
Caregiver status^e^	1.04 (0.75-1.44)	.82	1.09 (0.75-1.60)	.64	1.11 (0.75-1.65)	.61

Abbreviations: AOR, adjusted odds ratio; NA, not applicable; OR, odds ratio.

^a^ Baseline characteristics were self-reported in the survey. All analyses applied survey weights. The impairments group was defined as respondents who identified as disabled, who reported a decline in health and activity, and/or who replied yes to any of the 6 functional limitation questions derived from the American Community Survey. Among respondents with impairments, subgroups of disability identity status were determined by yes vs no responses to the question, “Do you have a disability?”

^b^ Adjusted logistic regression model 1 controlled for age category, gender, race, ethnicity, education level, household income, employment status, marital status, caregiver status, and veteran or active military status.

^c^ Adjusted logistic regression model 2 controlled for the same covariates as model 1 with the addition of covariates for impairment number (dichotomized as ≤3 vs ≥4) and impairment types that were imbalanced across groups (decline in health and activity; mobility; psychological, memory, or cognition; dressing and bathing; and errands).

^d^ The other racial category consisted of respondents who selected Asian (n = 49), multiple race options (n = 68), or other (n = 34). These categories were collapsed due to small cell size across groups.

^e^ Caregiver status was defined as respondents who indicated being very or fairly involved in someone else’s health care, such as through medical decision-making, care coordination, appointment-related transportation, provision of physical assistance, and billing and paperwork.

## Discussion

In this cross-sectional analysis of MCSIC’s nationally representative survey of US adults, we found a striking misalignment between standard functional inquiries (ACS-6) and a person’s identification with disability or their experience of a decline in health and activity. Nearly 1 in 4 participants who said yes to having a disability responded no to the entire set of ACS-6 questions and, thus, would have been missed by the assessment’s measure of function alone. Similarly, most respondents in the impairment group did not identify as disabled but still would have been labeled as having a disability according to their ACS-6 responses. This group also had many individuals with a reported limitation in work or activity that the ACS-6 failed to capture.

These findings add to the existing body of literature about the inadequacies of current disability measures.^20,24,37^ For example, 1 study found that the ACS-6 failed to identify 20% of impairments and disabilities as captured in the National Survey on Health and Disability (NSHD).^20^ Along with the ACS-6, the NSHD uses open-ended descriptions and 7 categories of medical conditions to characterize disabilities. Accordingly, the NSHD allows for comparisons between condition-based and function-based measures, but it does not have isolated assessments of disability identity or limitations to social participation; nor does it survey a nationally representative population.

Our study adds to the literature by examining differences in procedural justice perceptions along this often forgotten dimension of diversity. Our adjusted models controlled for sociodemographic factors that are associated not only with identity formation but also exposure to systems of oppression or privilege. Therefore, our unadjusted results might better account for intersectionality, multidimensional identities, and lived experiences.

From the MCSIC survey, we found that participants with impairments generally had worse perceptions of trust, communication, respect, and fairness in their health care encounters both before and after controlling for sociodemographic traits. Many also reported that they almost always had lingering uncertainty about the care plan after their health care encounters. Given the competing demands for clinicians’ time and attention, this finding is not surprising. Such systemwide barriers to communication may be exacerbated for some people with disabilities. Some physicians use strategies to improve communication for patients with impaired vision, hearing, or cognition that are misaligned with patients’ preferences and Americans With Disabilities Act mandates (eg, yelling or writing notes on paper instead of using American Sign Language interpreters).^38^

In both crude and adjusted models, participants with impairments who identified as disabled gave lower ratings of fairness and serious illness care but reported higher levels of confidence to speak up or ask questions during visits compared with those who did not identify as disabled. Additionally, the group that identified as disabled gave higher crude ratings of clinicians’ effort and appreciation of patients’ health goals.

To date, much of our understanding about disability identity has either come from qualitative research reflecting individual experiences or from analyses of scales designed to assess the formation of a disability identity.^28^ Forber-Pratt et al^28^ described disability identity development as a “fundamentally social process…formed through mirroring, modeling, and recognition.”^(p198)^ Health professionals contribute to this social process, especially in relation to accepting, rejecting, or stigmatizing bodily functions and appearances.

Common themes that have emerged from the existing literature on disability identity may be reflected in our results. First, disability identification is believed to enhance self-efficacy, self-worth, and a sense of community and acceptance, all of which could mitigate internalized ableism.^39,40,41^ Disability identification is also thought to promote disability activism and role modeling.^39,42,43^ Taken together, these themes could explain why, in our study, the participants who identified as disabled had favorable perceptions about their clinicians’ intentions or efforts while also having unfavorable perceptions of fairness and serious illness care. Their disability identification may have corresponded with greater recognition of discrepancies between their ideal and their actual care experiences.

In the literature, few studies have investigated factors associated with disability identification, and many of the studies that do exist have failed to document participants’ race or ethnicity.^28^ In our study, we not only examined procedural justice perceptions but also explored trends in the adoption of a disability identity across multiple sociodemographic attributes. We found that the odds of adopting a disability identity varied by racial category but only in crude models, whereas Hispanic ethnicity and female vs male gender were nonsignificant in all identity models.

In contrast, disability identity was strongly associated with unemployment and retirement in both crude and adjusted models. It is possible that these participants were exposed to the term disability as a binary characterization of their health status or benefit qualifications while filing for disability or retirement. An alternative hypothesis aligns with Garland Thomson’s^44^ observation that “nowhere is the disabled figure more troubling to American ideology and history than in relation to the concept of work.”^(p46)^ Employees could be reluctant to espouse a disability identity in a workplace culture that celebrates efficiency and capabilities.^45^ Additionally, discriminatory hiring practices may be at play; the prevalence of disabled persons (ACS-6 definition) without a job who were available and seeking work was twice that of the nondisabled population in 2022, and these rates were highest for Black and Hispanic respondents with disabilities.^10^

Annual household income was also strongly associated with disability identification in crude and adjusted models. Compared with participants with impairments in the lowest income category (<$30 000 per year), those earning more than $100 000 per year had significantly lower odds of reporting that they had a disability. This finding may be due to “othering” (ie, dissociation from disability) or social desirability bias, though it is also likely that people with higher incomes are able to access more or better accommodations that reduce environmental barriers to full participation in daily life.^26^

### Limitations

This study has several limitations and unobserved variables. First, we used a static, binary measure of disability identification rather than a dynamic scale. Second, the survey format did not lend itself to understanding the interplay among impairments, disabilities, identities, and procedural justice perceptions. Third, duration of impairment and number of health care encounters were not captured. A fourth limitation is the collapsed racial category, the lack of information about sexual identities, and the exclusion of gender-expansive groups. The intersectionality of disability, gender, and sexual identity remains an understudied topic, but several reports have suggested that sexual and gender minority groups with disabilities are more affected by disability and barriers to care compared with heterosexual and/or cisgender adults.^46,47,48,49,50^ Fifth, the COVID-19 pandemic disrupted in-person recruitment to the AmeriSpeak panel. However, a representative sample was achieved for the MCSIC survey, and participants were unaware of an analytic interest in disability at the time of survey recruitment and completion.

## Conclusions

In this cross-sectional survey study of US adults, we found that people with functional impairments do not necessarily identify as disabled, and vice versa. We also observed that perceptions of fairness, trust, and respect differed between these groups. Administrative databases, public health surveillance systems, and health records should improve their capture of disability information, and our findings suggest the value of asking about disability identity alongside measures of function. More complete data will inform policies to mitigate health disparities for people with impairments and for those who identify as disabled.

## Notes

Supplement 1. **eMethods.** Survey Design and Sampling Method **eFigure 1.** Derivation of Study Population **eTable.** Original Wording of Procedural Justice Questions in the MCSIC Survey **eFigure 2.** Dichotomized Procedural Justice Ratings by Disability Identity (DAI), Adjusted for Sociodemographic Traits and Number/Type of Impairments
eReferences

Supplement 2.
Data Sharing Statement
