# Errors in Table 2

**DOI:** 10.1001/jamahealthforum.2023.4048

**Published:** 2023-11-03

In the Original Investigation titled “Impairment and Disability Identity and Perceptions of Trust, Respect, and Fairness,”^1^ published on September 22, 2023, in Table 2, the crude *P* value for age ≥75 years was missing a decimal place; additionally, some “NA” and “1 [Reference]” labels had been mistakenly transposed across some cells in the odds ratio and *P* value columns. This article has been corrected.^1^
