# Supplementary material for: Impairment and Disability Identity and Perceptions of Trust, Respect, and Fairness
Source: JAMA Health Forum. 2023 Sep 22;4(9):e233180. doi: 10.1001/jamahealthforum.2023.3180 (PMC10517379; doi:10.1001/jamahealthforum.2023.3180)
Supplement: Supplement 1. — eMethods. Survey Design and Sampling Method eFigure 1. Derivation of Study Population eTable. Original Wording of Procedural Justice Questions in the MCSIC Survey eFigure 2. Dichotomized Procedural Justice Ratings by Disability Identity (DAI), Adjusted for Sociodemographic Traits and Number/Type of Impairments eReferences [file jamahealthforum-e233180-s001.pdf]

Supplemental Online Content

Salinger MR, Feltz B, Chan SH, et al. Impairment and disability identity and perceptions of trust, respect, and fairness. *JAMA Health Forum*. 2023;4(9):e233180. doi:10.1001/jamahealthforum.2023.3180

eMethods. Survey Design and Sampling Method

eFigure 1. Derivation of Study Population

eTable. Original Wording of Procedural Justice Questions in the MCSIC Survey

eFigure 2. Dichotomized Procedural Justice Ratings by Disability Identity (DAI), Adjusted for Sociodemographic Traits and Number/Type of Impairments

eReferences

This supplemental material has been provided by the authors to give readers additional information about their work.

© 2023 Salinger MR et al. *JAMA Health Forum.*

eMethods. Survey Design and Sampling Method

The Massachusetts Coalition for Serious Illness Care (MCSIC) commissioned a survey in April-May of 2021 to learn about patient perceptions of their healthcare. The survey population was a nationally-representative sample of 1854 US adults (**eFigure 1**). The sample was obtained by NORC at the University of Chicago via their AmeriSpeak® panel, which uses multi-staged, area-based probability sampling to achieve 97% sample frame coverage for the residential U.S..[1] AmeriSpeak® panel recruitment took place via mail, telephone, and face-to-face field interviewers, though in-person recruitment was paused during the COVID-19 pandemic.[1] NORC reports two response rates for the AmeriSpeak® panel: across all years (2014-2021), the cumulative weighted household response rate (i.e., the portion who joined the panel among those who were invited) is 21.9%; across all recruitment years with non-response follow-up (2014-2018 and 2021), the cumulative weighted household response rate is 34.0%.[1] (Refer to Technical Overview of the AmeriSpeak® panel for additional sampling methods and response rate details.[1])

For the MCSIC survey, the following categories had a minimum enrollment target of 100:

- low income(<$50K/yr)
- low income + Black race
- low income+ Hispanic ethnicity
- age ≥65,
- "Yes" to ≥1of the 6 disability questions derived from the American Community Survey (ACS-6) or the disability identity question
- "Yes" to decline in health/activity over the past year + self-reported diagnosis of diabetes, asthma, lung disease, heart disease, cancer, dementia, chronic kidney disease, depression, anxiety, or other serious mental illness.

All AmeriSpeak® panelists had provided informed consent and completed an initial questionnaire regarding their basic household and demographic information.[2] Panelists who were invited to participate in the MCSIC survey were then given the following introduction to the survey topic:

"Today, AmeriSpeak® is conducting a survey with people about their health care experiences and perspectives, including people with serious illnesses as well as future thoughts about serious illnesses for everyone else. When you hear the term serious illness, it means a disease or health issue that makes you feel sick enough that it's increasingly hard to do your normal levels of work and activity (for example, conditions like cancer, heart disease or dementia). We want to learn about your experiences and include your opinions. All answers will be kept confidential and anonymous."

Survey data were collected in English or Spanish, either online or via phone. NORC controlled data quality by measuring survey completion times and monitoring for surveys with "straight-line" responses, frequent skips, inconsistent responses, and/or duplicates in recruitment.[3]
Of the 6126 AmeriSpeak® panelists invited to participate, 1854 (30.3%) completed the MCSIC survey.[4] The margin of error was ±3.1 percentage points, adjusted for design effect.[4] Survey weights ensured the study population was reflective of the US Census population.

NORC's IRB oversaw survey recruitment, administration, and informed consent. The survey instrument and analysis were reviewed by Harvard Longwood Campus IRB.

© 2023 Salinger MR et al. *JAMA Health Forum.*

eFigure 1. Derivation of Study Population

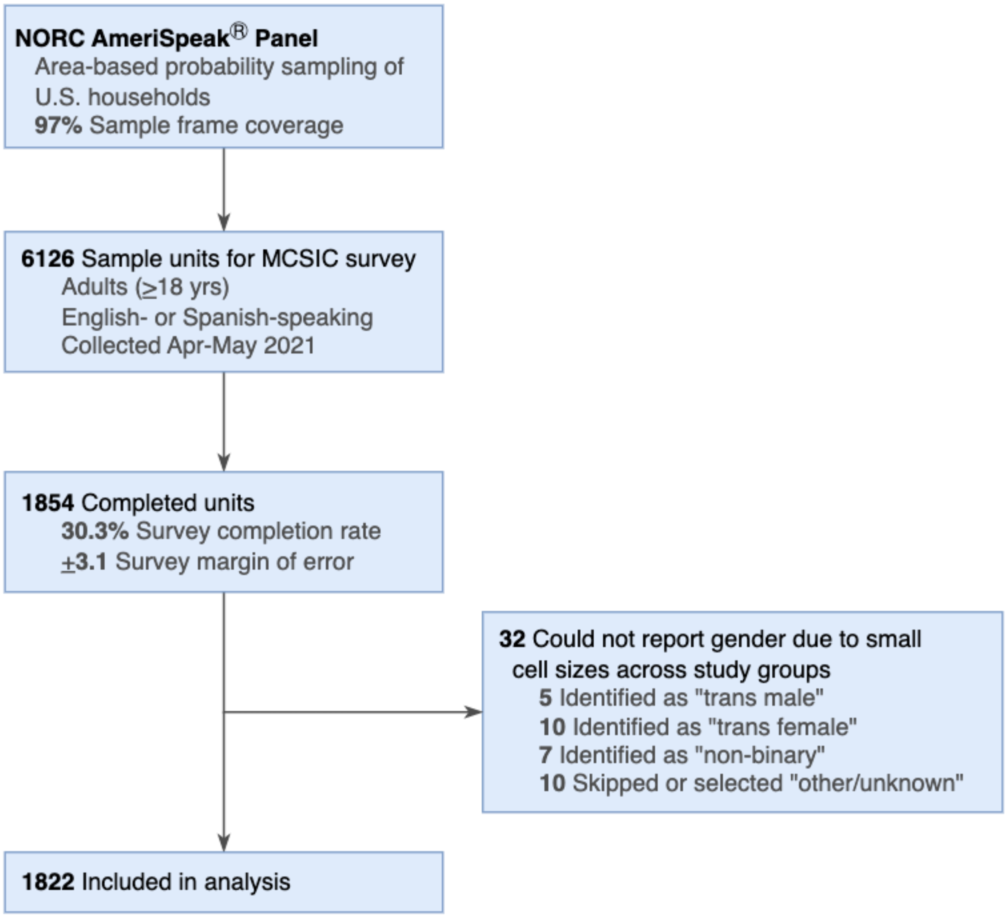

The Massachusetts Coalition for Serious Illness Care (MCSIC) commissioned a survey of U.S. adults (English- or Spanish-speaking) that was conducted in April-May of 2021.

The survey population was obtained from the AmeriSpeak® panel, managed by NORC at the University of Chicago. AmeriSpeak® uses a multi-staged probability-based sampling method through which they have achieved an estimated sample frame coverage of 97%.

Total households invited to participate in MCSIC survey = 6126; Total completed surveys= 1854 (30.3% of invited households); Margin of error = ±3.1 percentage points.

The current analysis excluded 32 respondents who indicated a gender other than "male" or "female" due to small cell size across study groups and risk of confounding if collapsed into a single gender category.

The resultant study sample consisted of 1822 English- or Spanish-speaking adults (age≥18).

See **eMethods** and/or Technical Overview of the AmeriSpeak® Panel[1] for additional details.

© 2023 Salinger MR et al. *JAMA Health Forum.*

eTable. Original Wording of Procedural Justice Questions in the MCSIC Survey

Original Wording of Questions	Answer Choices	Dichotomized*
Generally speaking, how much effort would you say doctors, nurses, and other health professionals make to help you understand your health issues? †	No effort, A little effort, Some effort, A lot of effort	A lot
Generally speaking, how much effort would you say doctors, nurses, and other health professionals make to include what matters most to you in making decisions about your care and treatments? †	No effort, A little effort, Some effort, A lot of effort	A lot
How often have you left a health care visit feeling unsure about your medicines, what was discussed, or what to do next?	Never, Rarely, Sometimes, Often, Always	Often, Always
Generally speaking, how well do you feel that doctors, nurses and other health professionals understand your health goals †	Not at all, Not very well, Fairly well, Very well, Don't know	Fairly/Very well
How often have you been afraid to ask questions, speak up, or disagree with doctors, nurses, and other health professionals because you're worried it may impact your care?	Almost none of the time, Some of the time, Most of the time, Almost all of the time, Don't know	Almost none of the time
How much of the time do you trust doctors, nurses, and other health professionals to do what is right for you? ‡	Almost none of the time, Some of the time, Most of the time, Almost all of the time, Don't know	Almost all of the time
How much of the time do doctors, nurses, and other health professionals treat you with dignity and respect? ‡	Almost none of the time, Some of the time, Most of the time, Almost all of the time, Don't know	Almost all of the time
How often do doctors, nurses, and other health professionals talk down to you or make you feel inferior? ‡	Almost none of the time, Some of the time, Most of the time, Almost all of the time, Don't know	Almost none of the time
Was there a time in the last 12 months when you felt that you were treated unfairly by doctors, nurses, or other health professionals? ‡	Yes, No	Yes
How would you rate the US health care system when it comes to meeting the needs of people with serious illnesses? §	Excellent, Good, Fair, Poor, Not sure	Good, Excellent

*Survey answer choices were transformed from Likert scale format to binary variables, defined as the listed answer choice(s) vs. all other answer choices. These dichotomizations were made according to the distribution of responses and the their valuative meanings.

†Adapted from CollaboRATE, a 3-item validated scale used to assess shared decision making in clinical settings[5]

‡Adapted from Kaiser Family Foundation's "Undefeated Survey on Race and Health"[6]

§Adapted from Kaiser Family Foundation's survey of "Serious Illness in Late Life: The Public's Views and Experiences"[7]

© 2023 Salinger MR et al. *JAMA Health Forum.*

eFigure 2. Dichotomized Procedural Justice Ratings* by Disability Identity (DAI),† Adjusted for Sociodemographic Traits and Number/Type of Impairments‡

Adjusted Odds of Response*‡

Multivariable Logistic Regression Models

	Yes-DAI vs. No-DAI†	aOR (95%CI)	p-value§
1. A lot of clinician effort to help me understand health issues		0.96(0.66,1.41)	0.84
2. Clinicians understand health goals very / fairly well		1.03(0.69,1.55)	0.88
3. A lot of clinician effort to include what matters most		1.04(0.71,1.50)	0.86
4. Often / always left visit unsure about discussion or next steps		1.27(0.80,2.01)	0.32
5. Almost never afraid to ask questions or disagree		1.36(0.92,2.02)	0.12
6. Almost always trust doctors to do what's right		1.09(0.74,1.61)	0.67
7. Almost always treated with dignity & respect		0.96(0.66,1.39)	0.82
8. Almost never felt inferior or talked down to		0.90(0.61,1.33)	0.59
9. Have been treated unfairly by clinicians in past year		1.91(1.27,2.89)	<0.01
10. US healthcare system serious illness care is good / excellent		0.52(0.35,0.77)	<0.01

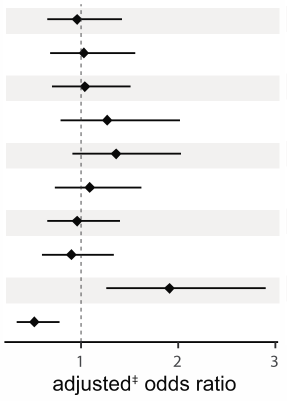

*Responses to survey questions about procedural justice perceptions – i.e., perceived trust, communication, respect, and fairness – were transformed from 5-point Likert scale measures to binary outcomes, according to the distribution of responses and the their valuative meaning.

†The group with impairments was defined as respondents who identified as disabled, who reported a decline in health and activity, and/or who replied "yes" to any of the six functional limitation questions derived from the American Community Survey (ACS-6). Among those with impairments, subgroups of disability identity (DAI) status were determined by "yes" vs. "no" responses to the question, "do you have a disability?"

‡Logistic regression models adjusted for age category, gender, race, ethnicity, education level, household income, employment status, marital status, caregiver status, veteran or active military status, number of impairments (dichotomized as $\leq$3 vs. $\geq$4 due to small sample size), and types of impairment. Types of impairment were included as indicator variables for health/social decline, errands, psych/cognition, dressing/bathing, and mobility. Hearing and vision impairments were already balanced across groups of disability identity and thus were excluded from the models. All covariates were based on survey self-report.

§ p-value derived from Wald test.

All analyses applied survey weights.

Whiskers represent 95% confidence intervals.

Among those with impairments, the number of survey participants who skipped each question is 1, 6,1, 0, 1, 3, 2, 5, 0, and 0 for questions 1-10, respectively.

© 2023 Salinger MR et al. *JAMA Health Forum.*

eReferences

1. AmeriSpeak Technical Overview 2019 02 18.pdf. Accessed February 28, 2023. https://amerispeak.norc.org/content/dam/amerispeak/research/pdf/AmeriSpeak%20Technical%20Overview%202019%2002%2018.pdf

2. DOCUMENTATION FOR NORC'S AMERISPEAK® PANEL FOR INSTITUTIONAL REVIEW BOARDS. Published online May 15, 2021. Accessed June 30, 2023. https://amerispeak.norc.org/content/dam/amerispeak/supporting-documents/NORC%20AmeriSpeak%20Information%20for%20IRBs%202016%2010%2018.pdf

3. ESOMAR 28. 28 Questions to Help Research Buyers of Online Sample. Accessed June 24, 2023. https://amerispeak.norc.org/us/en/amerispeak/about-amerispeak/esomar.html

4. *Serious Illness Care 2021*. AmeriSpeak & American Association for Public Opinion Research; 2021:2.

5. Barr PJ, Forcino RC, Thompson R, et al. Evaluating CollaboRATE in a clinical setting: analysis of mode effects on scores, response rates and costs of data collection. *BMJ Open*. 2017;7(3):e014681. doi:10.1136/bmjopen-2016-014681

6. KFF/The Undefeated Survey on Race and Health. Published online 2020.

7. DiJulio B, Hamel L, Wu B, Brodie M. Serious Illness in Late Life: The Public's Views and Experiences.

© 2023 Salinger MR et al. *JAMA Health Forum.*
