# Supplementary material for: Impairment and Disability Identity and Perceptions of Trust, Respect, and Fairness
Source: JAMA Health Forum. 2023 Sep 22;4(9):e233180. doi: 10.1001/jamahealthforum.2023.3180 (PMC10517379; doi:10.1001/jamahealthforum.2023.3180)
Supplement: Supplement 2. — Data Sharing Statement [file jamahealthforum-e233180-s002.pdf]

Data Sharing Statement

Salinger. Impairment and Disability Identity and Perceptions of Trust, Respect, and Fairness. *JAMA Health Forum*. Published September 22, 2023. doi:10.1001/jamahealthforum.2023.3180

Data
Data available: No

Additional Information
Explanation for why data not available: This patient-related information is not available for sharing. Upon request, a codebook can be made available to replicate analyses.
